# 886. The Impact of the COVID-19 Pandemic on Clinical Follow-Up, Monitoring and Regimen Discontinuation for People Living with HIV in the US

**DOI:** 10.1093/ofid/ofab466.1081

**Published:** 2021-12-04

**Authors:** Gerald Pierone, Jennifer S Fusco, Laurence Brunet, Cassidy Henegar, Jean A van Wyk, Supriya Sarkar, Vani Vannappagari, Andrew Zolopa, Michael B Wohlfeiler, Gregory Fusco

**Affiliations:** 1 Whole Family Health Center, Vero Beach, FL; 2 Epividian, Inc., Durham, NC; 3 ViiV Healthcare, Research Triangle Park, NC; 4 AIDS Healthcare Foundation, Miami, FL

## Abstract

**Background:**

The COVID-19 pandemic has disrupted health care services for people living with HIV (PLWH). This study aimed to compare rates of clinical visits, viral load monitoring and antiretroviral therapy (ART) regimen discontinuation among virally suppressed PLWH in the US before and during the COVID pandemic.

**Methods:**

The study population consisted of ART-experienced PLWH ≥18 years of age and active in care in the OPERA cohort within 2 years prior to 31OCT2020. Virally suppressed PLWH (i.e., viral load < 200 copies/mL) were included if they switched to either dolutegravir/lamivudine or a dolutegravir- or bictegravir-based 3-drug regimen between 01MAY2019 and 30APR2020. The study periods spanned from 01MAY2019 to 28FEB2020 (pre-COVID) and 01MAR2020 to 31OCT2020 (during COVID). Incidence rates of clinical visits, viral load measurements and regimen discontinuation were estimated using univariate Poisson regression for both study periods. In-person visits comprised any scheduled or walk-in outpatient, inpatient, emergency or laboratory visit. Telehealth visits comprised any phone or video encounters.

**Results:**

The study included 4806 PLWH in the pre-COVID and 4992 in the COVID period. Rates of in-person visits were reduced almost 2-fold during COVID, while telehealth visits increased almost 9-fold, resulting in an overall reduction in any visits rates from 10.07 visits per person-year (95% CI: 9.93, 10.21) pre-COVID to 7.10 (95% CI: 7.01, 7.19) during COVID [Fig 1]. Rates of viral load measurements dropped from 2.99 viral loads per person-year (95% CI: 2.92, 3.07) pre-COVID to 1.97 (95% CI: 1.92, 2.02) during COVID [Fig 2]. Regimen discontinuation rates were also reduced from 14.3 discontinuations per 100 person-years pre-COVID (95% CI: 12.7, 16.1) to 9.6 (95% CI: 8.6, 10.8) during COVID [Fig 3]. In both study periods, virologic failures were detected in < 1% of PLWH with ≥ 1 viral load.

Figure 1. Incidence rates for overall, in-person, and telehealth visits during the pre-COVID (open circle) and the COVID (filled circle) study periods

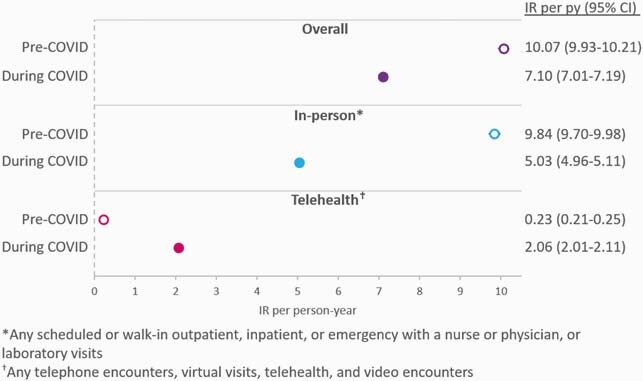

Incidence rates for viral load measurements during the pre-COVID (open circle) and the COVID (filled circle) study periods

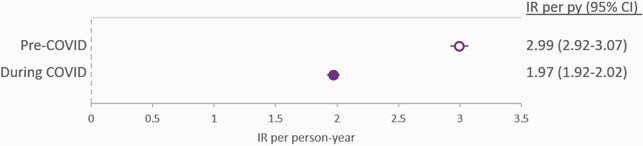

Incidence rates for regimen discontinuation during the pre-COVID (open circle) and the COVID (filled circle) study periods

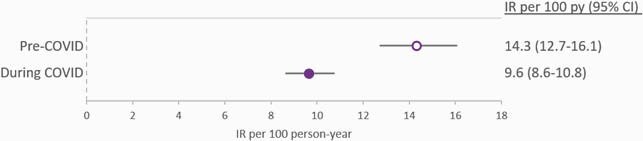

**Conclusion:**

The COVID pandemic has led to an important reduction in the frequency and type of clinical follow-up visits and viral load monitoring among virally suppressed PLWH in the US. A reduction in regimen discontinuation rates was also observed, presumably associated to less frequent follow-up. The long-term impact of the pandemic on HIV care remains uncertain.

**Disclosures:**

**Gerald Pierone, MD**, **Epividian** (Board Member) **Jennifer S. Fusco, BS**, **Epividian, inc** (Employee) **Laurence Brunet, PhD**, **Epividian, inc** (Employee) **Cassidy Henegar, PhD**, **GSK** (Shareholder)**ViiV Healthcare** (Employee) **Jean A. van Wyk, MB,ChB**, **GlaxoSmithKline** (Shareholder)**ViiV Healthcare** (Employee) **Supriya Sarkar, PhD**, **GSK** (Shareholder)**ViiV Healthcare** (Employee) **Vani Vannappagari, MBBS, MPH, PhD**, **ViiV Healthcare Limited** (Employee) **Andrew Zolopa, MD**, **GlaxoSmithKline** (Shareholder)**ViiV Healthcare** (Employee) **Michael B. Wohlfeiler, MD**, **Epividian, inc** (Board Member)**ViiV Healthcare** (Research Grant or Support) **Gregory Fusco, MD, MPH**, **Epividian, inc** (Employee)

